# Biochemical analysis in congenital neuroblastoma

**DOI:** 10.1515/almed-2022-0112

**Published:** 2022-12-16

**Authors:** Cristina Montero Domínguez, Alicia Ortiz Temprado, Laura Martínez Figueras, Alba Guillamón Seoane, Miguel Fernández Ruano

**Affiliations:** Service of Clinical Biochemistry, Hospital General Universitario Gregorio Marañón, Madrid, Spain

**Keywords:** catecholamine/creatinine index, catecholamines, congenital neuroblastoma, fractionated metanephrines, homovanillic acid, vanillylmandelic acid

## Abstract

**Objectives:**

The incidence of congenital neuroblastoma has increased in the recent years. The purpose of this study was to describe the clinical and biochemical characteristics of cases of congenital neuroblastoma diagnosed in our center.

**Case presentation:**

We report three cases of congenital neuroblastoma diagnosed in our hospital. In two, diagnosis was made prenatally, whereas the other case was detected in the immediate neonatal period. In the three cases, neuroblastoma was located in the abdominal region and exhibited elevated concentrations of catecholamines or their metabolites in single voided urine samples. Two tumors were classified as stage M, and one as stage L2. The *N-MYC* oncogen was not amplified in any of the cases studied. Histopathological analysis was favorable in the three cases. The tumor was resected in two patients. The three received chemotherapy.

**Conclusions:**

The measurement of catecholamines and their metabolites is essential in the diagnosis of neuroblastoma. When 24 h urine cannot be collected, single voided urine can be used to calculate the index based on creatinine concentrations.

## Introduction

Neuroblastoma (NBL) is the most frequent extracranial solid tumor in childhood, with an incidence of 10 cases per million subjects younger than 15 years. NBL accounts for 7% of all pediatric tumors, and it causes 15% of cancer-related deaths in children [1]. It originates in neural crest cells and can be located anywhere along the neuroectodermal sympathetic nervous chain. Symptoms depend on the location of the primary tumor and the presence of paraneoplastic or metastatic syndromes. As many as 90% are catecholamine-producing tumors; therefore, diagnosis is based on biochemical findings (determination of catecholamines and their metabolites) coupled with imaging studies and histological analysis [1, 2].

Congenital NBL is defined as neuroblastoma detected during the prenatal period or within the immediate 28 post-birth days, accounting for 5% of all NBLs [2]. In the case of NBLs diagnosed prenatally, more than 90% originate in the adrenal medulla [3, 4]. Hence, a solid adrenal mass on prenatal ultrasound is suggestive of congenital NBL. In these cases, it is necessary to perform a differential diagnosis with other entities, such as hydronephrosis, adrenal hemorrhage, and infradiaphragmatic extralobar pulmonary sequestration [2, 3]. The detection of congenital NBLs has grown as a result of the increasing use of prenatal ultrasound in the recent times. Therefore, it is important to improve knowledge on the management of this condition in newborns.

## Case presentation

We report three cases of congenital NBL diagnosed in our center between 2019 and 2020. Table 1 summarizes the characteristics of each case.

**Table 1:** Summary of reported cases of congenital NBL.

Case	Age at diagnosis	Primary tumor site	Metastasis sites	Elevated catecholamines or metabolites	*N-MYC* amplification	Histopathology	Stage at diagnosis	Treatment
A	37 GW	Adrenal	Bones and liver	NA/creatinine	No	Favorable	Medium-risk M	Surgery and chemotherapy
				NMN/creatinine				
				HVA/creatinine				
				VMA/creatinine				
B	1 day of life	Adrenal	–	NMN/creatinine	No	Favorable	Low-risk L2	Chemotherapy
				HVA/creatinine				
				VMA/creatinine				
C	34 GW	Adrenal	Liver, retroperitoneal, subcutaneous, and muscle	NMN/creatinine	No	Favorable	Medium-risk M	Chemotherapy and surgery
				HVA/creatinine				
				VMA/creatinine				

GW, gestational week; NA, noradrenaline; NMN, normatenephrine; VMA, vanillylmandelic acid; HVA, homovanillic acid.

### Case A

An adrenal mass was identified on ultrasound in the fetus of a 29 year-old woman (3 pregnancies, 0 abortions) at 37 weeks of pregnancy. Magnetic resonance imaging (MRI) confirmed the presence of a tumor measuring 2.8 × 2.8 × 3.6 cm in the right adrenal region suggestive of NBL. Delivery was eutocic at 40 + 6 weeks of pregnancy and a boy was born with Apgar 9/10 and 3,360 g of weight. Postnatal ultrasound confirmed the presence of a mass measuring 5 × 3.3 × 3.6 cm.

**Figure 1:**
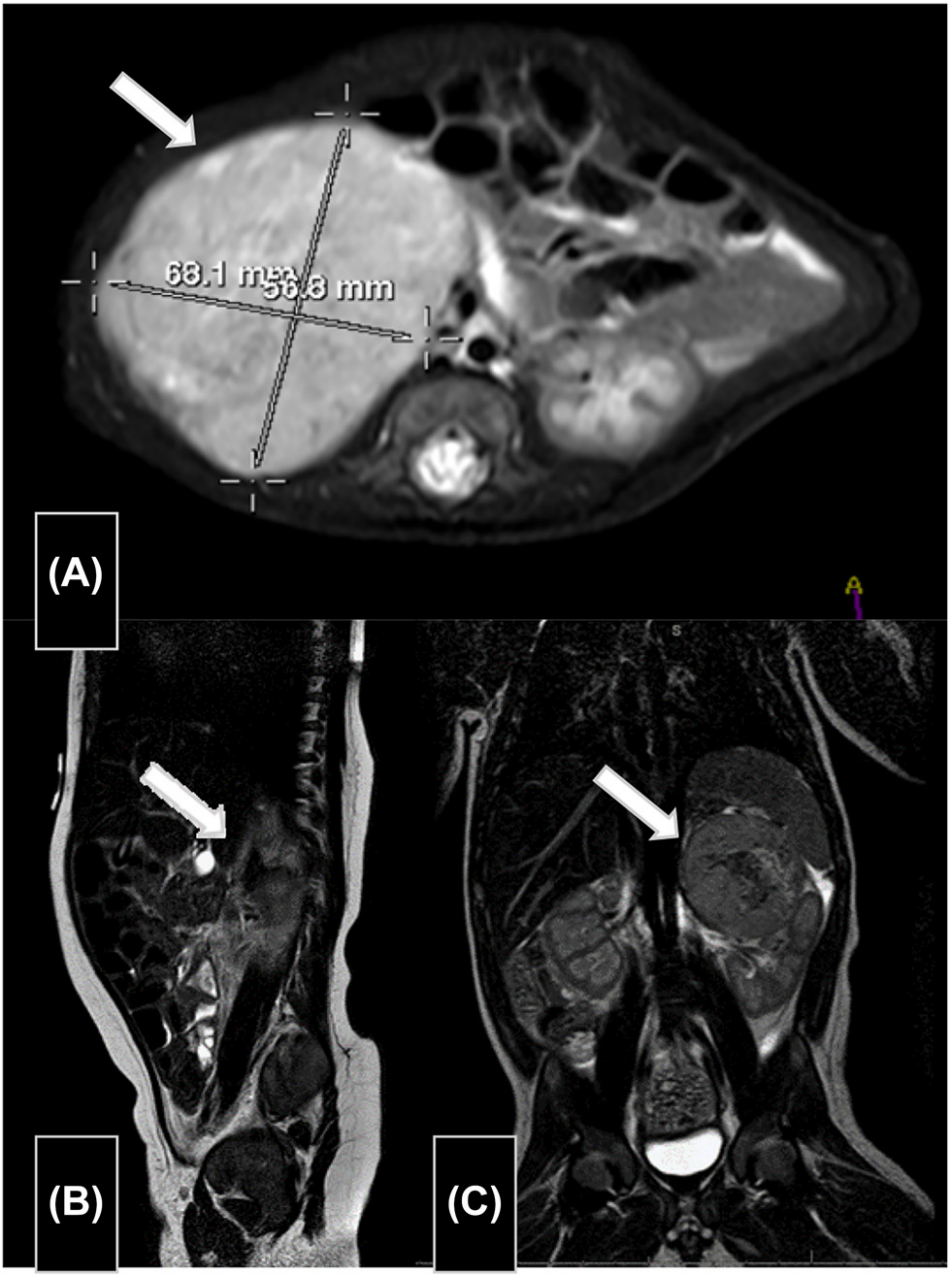
Abdominal magnetic resonances (A) Case A: mass of 6.8 × 5.7 × 7.6 cm in the right adrenal region. It abuts the inferior vena cava and the right renal vein and artery, as well as the right kidney, which is deformed and displaced inferiorly, and the right hepatic lobe. (B) Case B: solid mass in the right adrenal area of 3.6 × 2.6 × 5.1 cm. (C) Case C: left adrenal mass of 4.4 × 4.2 × 4.0 cm.

Single voided urine samples were collected for 3 days to determine concentrations of catecholamines and their metabolites by high-resolution liquid chromatography (HPLC) on an Agilent^®^ 1100 (Agilent Technologies, Santa Clara, USA) system using commercially available Recipe^®^ kits (RECIPE Chemicals + Instruments GmbH, Múnich, Germany). Elevated levels were observed for norepinephrine (NA)/creatinine; normetanephrine (NMN)/creatinine; homovanillic acid (HVA)/creatinine; and vanillylmandelic acid (VMA)/creatinine ratios (Table 2). At one month post-birth, abdominal MRI revealed that the right adrenal mass had grown and measured 6.8 × 5.7 × 7.6 cm (Figure 1A). Scintigraphy with ^131^I-meta-iodobenzylguanidine (MIBG) confirmed the presence of pathological chromaffin cells within the mass, with multifocal bone and liver involvement.

**Table 2:** HPLC-based biochemistry of catecholamines and their metabolites.

	Reference range	Case A	Case B	Case C
A, nmol/mmol creatinine	<42.6 ^10^	18.3 (10.1)	–	28.0 (13.0)^b^
NA, nmol/mmol creatinine	<182 ^10^	**802. 0 (163.0)**	–	76.7 (21.9)^b^
DA, nmol/mmol creatinine	<1975 ^10^	809 (40.8)	–	866.6 (6.3)^b^
MN, nmol/mmol creatinine	50–400 ^11^	235.1 (83)	161.8 (81.3)	83.2 (36.9)
NMN, nmol/mmol creatinine	590–1520 ^11^	**21745.6 (7335.4)**	**6658.9 (4627.5)**	**1971.3 (119.6)**
3-MT, nmol/mmol creatinine	Not available	2570.6 (647.0)	975.0 (722.2)	514.4 (94.1)
VMA, µmol/mmol creatinine	<10.8 ^10^	**115.2 (9.5)**	**35.6 (14.9)**	**43.6 (5.3)** ^**b**^
HVA, µmol/mmol creatinine	<21.7 ^10^	**144.1 (44.9)**	**36.4** ^**a**^	**60.9 (3.7)** ^**b**^

Results are expressed as mean values (standard deviation), with values exceeding reference range shown in bold. ^a^Data is only available for 1 day. ^b^Data is available for 2 days. A, adrenaline; NA, noradrenaline; DA, dopamine; MN, metanephrine; NMN, normatenephrine; 3-MT, 3-methoxytyramine; VMA, vanillylmandelic acid; HVA, homovanillic acid.

Treatment was initiated with alpha- and beta-blockers due to the hypertensive crisis suffered by the patient, to perform the surgical mass resection. Resection was performed by laparotomy at two months of life. Histopathological analysis revealed a poorly-differentiated NBL with a low mitosis-karyorrhexis index (MKI). Genetic analysis ruled out both *N-MYC* oncogene amplification and chromosome 11q deletion. The tumor was categorized as a medium-risk stage M NBL (International Neuroblastoma Risk Group Staging System (INRGSS)). Subsequently, the patient received chemotherapy (etoposide/carboplatin).

### Case B

A newborn with a prenatal diagnosis of Noonan syndrome, with Apgar 8/10 and a weight of 3,050 g. A right-sided adrenal mass was identified on ultrasound, consistent with congenital NBL. The analysis of single voided urine revealed elevated levels of catecholamine metabolites, especially for the NMN/creatinine ratio (Table 2). MRI confirmed the presence of a solid mass in the right adrenal region measuring 3.6 × 2.6 × 5.1 cm, consistent with the suspected diagnosis (Figure 1B). Scintigraphy with ^131^I-MIBG revealed the presence of pathological chromaffin cells at the level of the right adrenal gland, without other sites being involved.

Histopathological analysis revealed a poorly-differentiated NBL with a low MKI. Genetic analysis showed the absence of both *N-MYC* amplification and chromosome 11q deletion. The tumor was classified as low-risk stage L2 (INRGSS). The patient received chemotherapy (etoposide/carboplatin).

### Case C

A left-sided adrenal mass and agenesis of the corpus callosum were identified on ultrasound in the fetus of a 35 year-old woman (3 pregnancies, 2 abortions) at 34 weeks of pregnancy. Fetal MRI showed a tumor measuring 2.9 × 2.3 × 3.2 cm. Delivery was eutocic at 39 weeks of pregnancy and a boy was born with Apgar 9/10 and 3,130 g of weight. Postnatal ultrasound revealed a mass at the level of the left hypochondrium measuring 5.3 × 3.5 × 4 cm consistent with NBL.

Determination of catecholamines and their metabolites showed slightly elevated NMN/creatinine, HVA/creatinine, and VMA/creatinine ratios in serial single voided urine samples (Table 2). At 3 days post-birth, brain MRI confirmed prenatal diagnosis of complete agenesis of the corpus callosum with associated malformations. At 11 days of life, abdominal MRI (Figure 1C) confirmed the presence of NBL with liver, retroperitoneal, subcutaneous and muscle metastases. Histopathological analysis revealed a poorly-differentiated NBL with a low MKI. Genetic analysis ruled out both *N-MYC* oncogene amplification and chromosome 11q deletion. It was classified as medium-risk Stage M neuroblastoma (INRGSS).

At 1 month after birth, chemotherapy was initiated (etoposide/carboplatin), which reduced the size of the left adrenal mass, with a favorable response of liver, subcutaneous, and muscle lesions. At 4 months of life, tumor resection was performed.

## Discussion

Diagnosis of NBL is established based on the determination of catecholamines and their metabolites in urine, histophatological analysis and imaging studies [5]. Traditionally, VMA and HVA are most frequently used, with a sensitivity of 81.6 and 80.5%, respectively, with a better performance in the presence of metastatic disease (100% sensitivity, 99.7% specificity and an area under the curve (AUC) of 1 for the combination of HVA and VMA) [6]. There is evidence that the combination of NMN with VMA or HVA improves diagnostic performance, whereas the inclusion of 3-methoxytyramine increases diagnostic sensitivity to 95% [7], [8], [9]. Although secretion profiles are not generally provided in the case reports found in the literature, elevated levels of VMA and HVA have been reported in 33–38% of NBLs diagnosed prenatally [3, 4]. In our three cases, all patients had elevated concentrations of some of the analytes measured. This may be due to the larger size of the tumors described in this report, the measurement of a broader range of metabolites such as NMN or the fact that our results were adjusted for creatinine clearance.

A limitation to the analysis of catecholamine metabolites in urine in pediatric patients is the difficulty in obtaining 24 h urine samples. When 24 h urine cannot be collected, single voided urine can be used to calculate the index based on creatinine clearance. It should be taken into account that creatinine clearance is influenced by muscle mass and increases with height, which makes it necessary that an appropriate range of reference is established for each age [10, 11].

The International Neuroblastoma Risk Group developed the INRGSS to classify this type of tumors based on imaging studies previous to surgery. This group identified other prognostic factors associated with disease severity, namely, age at diagnosis (>18 months), histology, level of differentiation and cytogenetic and molecular characteristics [12]. None of our cases of congenital NBLs had unfavorable histopathological characteristics, i.e. *N-MYC* gene amplification or chromosomal aberrations.

NBL embraces clinically heterogeneous tumors with different behaviors. Correct diagnosis will determine the therapeutic approach, which may range from an expectant attitude to intensive chemotherapy and surgery [1, 2]. Early differential diagnosis – with a special role of urine catecholamine metabolite determination– is essential for an adequate therapeutic management.

## Key points

– Congenital NBL is a rare entity that should be considered in the presence of an adrenal solid mass on prenatal ultrasound.
– Upon prenatal suspicion of NBL, performing a biochemical analysis in the immediate postnatal period facilitates early diagnosis. Catecholamine metabolite determination in urine is one of the most reliable diagnostic methods.
– When 24 h urine cannot be collected, catecholamine metabolites can be determined through the analysis of a single voided urine sample by correcting metabolite values with creatinine and using reference values adjusted for age.
